# Impact of Tooth Loss and Other Risk Factors on Cognitive Impairment in Saudi Female Population

**DOI:** 10.1155/2019/6086515

**Published:** 2019-07-24

**Authors:** Atheer Abdulhade Ganem, N. C. Sandeepa, Afnan Hassan Alkhayri, Yosra Mohammed Mousa

**Affiliations:** ^1^Department of Orthodontics, College of Dentistry, King Khalid University, Abha, Saudi Arabia; ^2^DDS-Oral Biology, College of Dentistry, King Khalid University, Abha, Saudi Arabia; ^3^College of Dentistry, King Khalid University, Abha, Saudi Arabia

## Abstract

**Introduction:**

It is known that cognitive impairment is linked to aging and neurobiological, psychological, and social factors. Recently, however, mastication and the number of teeth has also attracted attention, with a previous case control study reporting a correlation between the loss of teeth and Alzheimer's disease.

**Objective:**

To investigate possible relationships between cognitive function and various demographic variables, stress, medical history, and number of natural teeth in a specified female population.

**Materials & Methods:**

A sample of the Saudi female population, 40–65 years of age, who visited the King Khalid University College of Dentistry (Abha, Saudi Arabia), was studied. Education, occupation, perceived stress, and medical history, along with the number of remaining teeth, were assessed. The Mini-Mental State Examination (MMSE) was used to assess cognitive performance and the results were statistically analyzed.

**Results:**

Subjects were divided into those with mild, moderate, and severe impairment based on MMSE score; the association between age, education, occupation, medical history, and cognitive function demonstrated statistically significant results. Fifty percent of subjects with 0–16 teeth exhibited severe cognitive impairment. Of the cognitive abilities, attention, recall, and language skills were linked to the number of remaining teeth. When subjects were categorized into only high and low cognitive impairment based on MMSE score, regression analysis did not reveal a significant correlation between any of the studied variables and cognitive impairment.

**Conclusion:**

Results of the present study add to the recent data and head towards the theory of likely connection between the number of teeth and hippocampus-dependent cognitive functioning. Results of regression analysis revealed an absence of conclusive relation in the latter part of study. Longitudinal analyses including comprehensive clinical dental data with brain-imaging will shed further light on probable causal relationship(s).

## 1. Introduction

Older adults usually present with some form of cognitive impairment. Mild cognitive impairment is a state representing the intermediate phase between normal aging and dementia and is also termed “predementia syndrome” [1]. In adults, the possibility of evolution of mild cognitive impairment to dementia is significantly higher than in individuals without cognitive impairment. Investigators based in the United States have reported that the annual progression from mild cognitive impairment to dementia occurs at a rate of 5.9%, which is significantly higher than the rate of progression of normal cognition to dementia, which is approximately 0.6% [2]. Thus, it is imperative to recognize risk factors for cognitive waning so as to delay the onset and deterioration of cognitive impairment.

Previous studies have identified possible risk factors for the development of dementia including inadequate education, depression, physical inactivity, poor dietary habits, and the presence of chronic diseases [3, 4]. Other possibilities that have emerged in the past decade as likely factors in the context of cognitive impairment include mastication and the number of teeth. Research involving animals and humans has proposed a probable causal connection between mastication and cognitive impairment [5].

Relatively few studies involving humans, however, have assessed whether cognitive problem is associated with the number of natural teeth [6, 7]. It is recognized that natural teeth and jaw movement give rise to sensory and motor feedback in the central nervous system [8]. Research has recently focused on determining the practical aspects of the theory of tooth loss and other risk factors with cognitive impairment and, thus, the preclinical stage of Alzheimer's disease (AD) and dementia in the Saudi female population.

To investigate our hypothesis, this community-based survey studied subjects based on MMSE score, dental examination, and various previously established factors of cognitive decline [9, 10]. Information regarding the number of remaining teeth, stress level, medical history, years of education, and occupation was collected. The purpose of this cross-sectional study was to explore the association between cognitive impairment and various factors, as well as the number of remaining teeth in an adult sample of the Saudi female population.

## 2. Materials and Methods

This study was approved by the Scientific Research Committee of King Khalid University, College of Dentistry (Abha, Saudi Arabia). Saudi females 40–65 years of age, who visited the King Khalid University College of Dentistry, were included in the study using a random sampling method. Before participation in the study, which was conducted over a period of 6 months, subjects were informed about the research and written informed consent was obtained. Variables including stress level, occupation, years of education, and any history of disease (cancer, cerebrovascular disease, myocardial infarction, diabetes mellitus, or hypertension) were recorded. Stress levels were recorded on Likert scale scored from 0 to 10 and categorized as follows: 0–3 (mild); 4–7 (moderate); and 8–10 (severe). Data regarding years of education (0–5, 6–10, 11–15, and 16–20 years) were recorded. Occupations were recorded and grouped accordingly. Assessment of cognitive mental status was performed using the Mini-Mental State Examination (MMSE), which is a widely used test for cognitive function, and includes tests of orientation, attention, memory, language, and visual-spatial skills. A score of 24–30 was classified as mild, 18–23 as moderate, and 0–17 was considered as severe impairment [11].

A thorough dental check-up was performed by two dentists under artificial lighting, and the number of teeth in each subject was documented, as being healthy, carious, completely erupted third molars, and treated teeth including crown, inlay, and abutment teeth for bridge. Root stumps and mobile teeth indicated for extraction were not included in the number of remaining teeth. The chi-squared test (test for significance) was applied at a 95% confidence interval (CI) to determine whether there was a relationship between cognitive performance and age, years of education, occupation, medical history, and remaining teeth and stress. In the latter part of the study, only 2 scales of cognitive impairment were used based on MMSE score and regression analysis was performed. An MMSE score ≥ 18 was classified as low cognitive impairment, and a score < 18 was classified as high impairment. The same variables were also tested using the 2 categories.

## 3. Results

The study included a sample of 300 females from the Saudi population. Subjects were categorized based on age group: 40–55 years (85.3% [n=256]) and 56–70 years (14.7% [n=44]). The mean (± SD) age of the study sample was 48.24 ± 6.53 years, with a minimum age of 40 and maximum of 70 years. Years of education were also assessed as part of the study: 26.3% of subjects had 0–5 years, 24% had 6–10, 32.3% had 11–15, and 17.3% reported 16–20 years. Among occupations, 81.7% of subjects reported none, while 16.3% were teachers, and 2% were physicians.

The frequency distribution of demographic variables, along with a comparative evaluation of these variables with MMSE score, is presented in Table 1. The majority (92.2%) of the 40–55 years' age group exhibited mild impaired cognitive function, while it was observed only in 7.8% of 56-70-year age group. Moderate impairment was observed in 69.3% of 40-55-year age group and 30.7% of 56-70-year age group. Severe cognitive impairment was observed in 87.5% of 40-55-year age group and 12.5% of 56-70-year age group. When years of education were considered and associated with MMSE score, severe impairment was observed in all subjects who reported 0–5 years of education. Moderate impairment was observed in 60.2% of individuals in this group. Among subjects with 6–10 years of education, 22.1% and 30.7% exhibited mild and moderate impairment, respectively. Severe cognitive issue was not observed in subjects with >5 years of education. Moderate impairment was observed in 30.7%, 6.8%, and 2.3% of subjects with 6–10, 11–15, and 16–20 years of education, respectively. Correlations between years of education and MMSE score are summarized in Table 1. Among those with 0–5 years of education, 100% exhibited severe impairment, whereas the majority (44.6%) of subjects with 11–15 years exhibited mild impairment. Correlation analysis between occupation and MMSE score revealed that physicians exhibited only mild cognitive impairment, whereas other groups exhibited distribution in all categories. Subjects were asked to report the perceived stress in their life and to score this stress from 0 to 10, with responses categorized as follows: 0–3 (mild); 4-7 (moderate); and 8–10 (severe). Mild cognitive impairment was observed among 47.5% of subjects with mild stress, 37.7% subjects with moderate stress, and 14.7% of subjects with high stress. Moderate impairment was seen in 56.8% subjects with mild stress, 33% of moderate stress, and 10.2% of high stress. Severe cognitive issue was observed in 75% of subjects with mild stress and 25% of subjects with high stress. Severe cognitive issue was not observed among subjects with moderate stress. On correlating medical history with MMSE score, mild impairment was seen in 83.3% of subjects with no medical history, 6.4% of hypertension, 5.4% of diabetes patients, 1% of CVS patients, 2.5% of hypothyroid, 1% of rheumatoid arthritis, and 0.5% of asthmatic patients. Moderate impairment was seen in 58% of subjects with no medical history, 12.5% of hypertensive, 19.3% of diabetes patients, 1.1% of CVS and hypothyroid patients, 4.5% of arthritis, and 3.4% of asthmatic patients. Severe impairment was noted among 75% of subjects with no medical history and 25% of subjects with hypertension. It was not observed among other categories of diseases. Based on the number of remaining teeth, subjects were classified as having 0–16 teeth, which is less than one-half the dentition, 17–28 teeth, and > 28 teeth. Mild impairment was seen in 2% of subjects with 0-16 numbers of teeth, 71.1% of subjects with 17-28 numbers of teeth, and 27% of subjects with more than 28 numbers of teeth. Moderate impairment was observed among 13.6% of subjects with 0-16 numbers of teeth, 81.8% of subjects with 17-28 numbers of teeth, and 4.5% of subjects with more than 28 numbers of teeth. Severe impairment was seen in 50% of subjects with 0-16 numbers of teeth and 17-28 numbers of teeth. No subjects with more than 28 teeth exhibited severe impairment (Table 1).

The MMSE questionnaire, which is divided into 5 sections, was used to assess cognitive function. Section 1 assessed orientation; 2 segments were measured, with a minimum score of 0 and a maximum score of 10. Total score was obtained, and overall scale of 2 points was structured. Those who scored 0–5 were categorized as having a low orientation and subjects scoring 6–10 score were classified as having a high orientation. Section 2 assessed registration; 1 element was measured, with a minimum score of 0 and a maximum score of 3. Total score was obtained, and overall scale of 2 points was structured. Subjects with a score of 0–1 were categorized as those with a low registration and those scoring 2–3 were categorized as those with a high registration. Section 3 assessed attention and calculation. One category was measured, and the minimum score was 0 and maximum score was 5. Total score was obtained and overall scale of 2 points was structured. Subjects with less attention were defined with a score of 0–2 score and a 3–5 score defined subjects with more attention. Section 4 included recall; 1 item was measured, with a minimum score of 0 and a maximum score of 3. Total score was obtained and overall scale of 2 points was structured. Those with poor recall were categorized with a score of 0–1 and a score of 2–3 characterized those with good recall. Section 5 was language and 6 items in the questionnaire were grouped and included under the language section, with a minimum score of 0 and a maximum score of 9. Total score was obtained and the overall scale of 2 points was structured. Those with poor language skill were categorized with a score of 0–4 and 5–9 categorized subjects with good language skills. Low orientation was demonstrated by 0.7% of subjects, 2% had low registration, while 23% had problems with attention and calculation. Thirty-two percent had poor recall and 1.3% had poor language skills. Orientation and registration did not demonstrate associations with any variables. Attention and calculation were related to age, years of education, occupation, medical history, and remaining teeth; however, stress did not play a role. Recall ability was related to age, years of education, occupation, and remaining teeth. Language skill was associated with years of education, stress, medical history, and remaining teeth.

Among the 40–55 years' age group, 91.3% exhibited more attention compared with only 8.7% of subjects in the 56–70 years' age group. The majority (90.2%) of 40-55-year-olds had good recall, while only 9.8% of 56-70-year-olds had good recall. Lower attention was noted in 58% of subjects with 0–5 years of education, while 31.9% with 6–10 years of education exhibited low attention. Only 4.3% of those with 11–15 years of education had attention issues and, among those with 16–20 years of education, 5.8% had low attention (Table 2). Poor recall was observed in 50% of subjects with 0–5 years of education, while only 21.9% of those with 6–10 years of education had poor recall issues. In the high-education groups, such as those with 11–15 and 16–20-years of education, 5.6% and 12.5% of subjects exhibited recall issues, respectively (Table 3). Attention and recall were also related to occupation. Low attention was observed in those with no reported occupation (95.7%), while 4.3% of teachers had low attention and doctors did not exhibit any issues. Poor recall was demonstrated by 92.7% of subjects with no reported occupation, while 6.3% of teachers had problems and only 1% of doctors had poor recall (Table 4). Attention and language skill were related to medical history. More attention was exhibited by 82.3% of those with no medical problems. Low attention was observed in 18.8% of hypertensive and 24.6% of those with diabetes mellitus. Poor language was observed in 50% subjects with hypertension, 25% of those with cardiovascular diseases, and 25% of those with hypothyroidism. Good language skill was observed in 76.7% of those without any medical issues (Table 5). Poor language skill was related to stress level. Seventy-five percent of subjects with severe stress levels had poor language skills, while good language skills were observed among 51.4% subjects with mild stress (Table 6).

Regarding the number of remaining teeth, among those with >28 teeth, only 4.3% had exhibited low attention, while 24.6% of those with less than one-half of their dentition had low attention. High attention was observed in 74.5% of subjects with 17–28 teeth. Only 1.3% of subjects with less than half dentation had more attention. Among those who had more than 28 numbers of teeth, 24.2% exhibited more attention. The results were statistically significant. P value was 0.000. In subjects with less than one-half of their dentition, only 3.4% had good recall, while 71.6% of those with 17–28 numbers of teeth had good recall. Those who had more than 28 numbers of teeth, 25% had good recall ability. Poor recall was observed among 78.1% of subjects having 17–28 numbers of teeth, while 8.3% of subjects with more than 28 numbers of teeth presented with poor recall. Statistically significant correlation (p value 0.000) was noted. Among subjects with >28 teeth, none had poor language skills. Language skill was correlated with number of remaining teeth. Poor language skill was observed among 50% of subjects among the subjects with less than half of dentation and group of subjects with 17–28 numbers of teeth. Among those who had more than 28 numbers of teeth, none presented with poor language issues. Among those with less than half of their dentition, only 6.1% demonstrated good language skills. Good language skill was demonstrated by 74.1% subjects with 17–28 teeth and 19.9% of subjects with more than 28 numbers of teeth. Statistically significant correlation was observed with number of remaining teeth to cognitive abilities of recall, attention, and language skill (Table 7). When cognitive ability of orientation was correlated with remaining number of teeth, poor orientation was observed only in subjects of 17–28 numbers of teeth, while none of subjects with less than half of dentation and more than 28 numbers of teeth presented with poor orientation. When considering high orientation, it was observed in 6.7% of subjects with less than half of dentition, 73.5% in those with 17–28 numbers of teeth, and 19.8% of subjects with more than 28 numbers of teeth. This correlation was not statistically significant (p value >0.05). Registration ability was calculated and compared with number of remaining teeth in the individual. Low registration was observed among 74.1% of subjects with 17–28 numbers of teeth. Only 6.5% of subjects with less than half of dentation had low registration issues. Among those had more than 28 number of teeth, 19.4% presented with low registration. High registration ability was observed in 33.3% of subjects with more than 28 numbers of teeth and 50% of subjects with 17–28 numbers of teeth. Among those who were having less than half of dentation, 16.7% had shown high registration ability. These results were not statistically significant (p value >0.05).

Multivariate and logistic regression analysis was also performed by dividing MMSE scores into high and low cognitive impairment categories to generalize the data. Low cognitive impairment was defined as MMSE score > 18 and high cognitive impairment was defined as an MMSE score ≤ 18. 97.3% of subjects exhibited low cognitive performance and 2.7% had high cognitive impairment. The chi-squared test was performed to analyze possible correlations among age, years of education, occupation, medical history, stress, and remaining teeth. Only the number of remaining teeth and years of education demonstrated a significant correlation with MMSE score in the context of low and high cognitive impairment. Other parameters such as age, occupation, medical history, and stress level did not show significant association with the two-grade system of MMSE score. The frequency distribution among the variables and correlations with MMSE score based on the two categories of cognitive impairment are shown in Table 8. Binominal logistic regression analysis was performed. Although the number of remaining teeth and years of education demonstrated relevance in the chi-squared test, no significance was observed with these variables in logistic regression analysis when the two scales were considered (Table 9).

## 4. Discussion

Illustrating the association between mastication and cognition is important in appraising risk factors for cognitive impairment in the elderly. It has been established that impairment of cognitive function is linked to aging, as well as neurobiological, emotional, and social factors [12, 13]. It has also been shown that artificial teeth (i.e., implant-supported reconstructions) can, to some degree, regenerate osseous perception in the central nervous system [14]. It is important to recognize risk factors for mild cognitive impairment so that intervention can be initiated as soon as possible to mitigate deterioration. Inadequate education, depression, chronic disease, lack of physical activity, and poor dietary habits have all been identified as probable risk factors in earlier studies [3, 4].

Several clinical and animal studies have demonstrated that mastication plays an active role in transferring sensory information to the brain and in sustaining learning and memory functions [15]. The hippocampus, an important region of the brain located in the medial-temporal lobe, is responsible for the formation and recovery of episodic memories in humans. Sensory signals from natural teeth and their adjacent periodontal mechanoreceptors are conveyed through the trigeminal sensory nerve to many areas in the central nervous system and, thus, influence hippocampal function [5]. Impairment in learning and spatial memory, along with neuron loss in the hippocampus, has been reported in animal studies after tooth extraction. Loss of functional molar teeth in rats has been shown to cause deteriorating and atypical variations in periodontal mechanoreceptors, consequential reduced sensory contribution from the periodontal ligaments, which in turn affects the morphology and function of neurons in the hippocampus [16, 17]. Based on previously reported data regarding a possible relationship between the oral cavity and hippocampal function collected from animal experimentation, data from similar studies involving humans is needed [18].

A previous population-based cross-sectional study demonstrated that edentulous subjects with artificial teeth performed significantly inferior in a variety of cognitive trials measuring several memory systems, especially episodic memory [19]. The results of that study, however, were founded exclusively on self-reports of tooth loss and tooth reconstruction(s). The aim of the present study, therefore, was to investigate the relationship between the number of natural teeth and various cognitive functions based on clinical examination and MMSE scores.

In 1975, Dr. Marshall Folstein developed the MMSE, and, since then, it has become a broadly used screening test for cognitive impairment and is regularly used as an inclusion/exclusion criterion and outcome measure in clinical trials. The test measures a variety of cognitive domains, including orientation to time and place, short- and long-term memory, registration, recall, constructional ability, language, and the ability to comprehend and follow commands [11].

It is important to score the test as fairly as possible in all subjects. Individuals with physical and/or noncognitive disabilities should not score lower simply because they are physically unable to perform specific tasks. In cases of education-related issues and, where the test cannot be modified, the task is omitted. If an item has been omitted due to physical disability, it is important to take this into account when scoring the test. The score from this task is subtracted from the total score (i.e., 30) to yield a new total. The individual's score is then adjusted according to this new total score.

In this research, we assessed the relationship between age, education, occupation, perceived stress, diseases, number of teeth, and performance on cognitive tests. Most similar studies have been based on self-reports of tooth loss and reconstructions and were performed in older age groups. Studies involving the Saudi population have shown that the incidence of tooth extraction among females is higher than in males in the 31–40 years' age group [20]. In the literature, there is little evidence of cognitive problems before 60 years of age [7]. This point of view, however, is not unanimously acknowledged [21]. Emerging consensus regarding the long gestation period of dementia proposes that adults < 60 years of age are expected to experience age-linked cognitive issue and that memory loss begins by approximately 45 years of age [22]. Based on this, we selected our study sample to be 40-65 years of age. The age at which cognitive impairment begins is imperative because interventions intended to modify cognitive ageing trajectories are more likely to be successful if they are applied when individuals first experience decline. The aim of the present study was, therefore, to investigate possible relationships between the number of natural teeth and cognitive functions by performing a clinical study with a focus on dental status. To our knowledge, this was the first such study in a Saudi population. The present cross-sectional clinical study was planned and performed to test the hypothesis that hippocampus-dependent cognitive measures are influenced by variation(s) in the number of natural teeth. All demographic parameters, including age, education, occupation, and medical history, were shown to have statistically differential percentage of samples exhibiting different levels of cognitive impairment. Stress levels were not found to be a statistically significant parameter in cognitive impairment in our research. When medical history and MMSE scores were analyzed, 83.3% of subjects with no medical history had only mild impairment. Relation of remaining number of teeth and cognitive impairment was a major point of focus in this research considering the theories and recent studies looking for a connection between both. Early part of statistics of the present research was leading to an association of these two factors. When considering the impairment score to mild, moderate, and severe scales, remaining teeth presented with statistically significant association along with other demographic parameters. Fifty percentage of patients with 0–16 number of teeth exhibited severe cognitive impairment. Attention, recall, and language skills were observed to have specific connotation with the number of remaining teeth, while orientation and registration ability seemed to be unrelated to the number of teeth present in an individual. To simplify the results, when MMSE score was divided into two categories (i.e., low and high cognitive impairment), the number of remaining teeth and years of education were still found to be correlated to cognitive function. However, in logistic regression analysis, these two variables were found to have no significant link with cognitive impairment. These findings shall be taken into contemplation for the further validation in future longitudinal projects.

## 5. Conclusion

When the two scales of low and high cognitive impairment were considered and logistic regression analysis was performed, remaining teeth variable has not shown a significant association with cognitive ability. Though in the univariate analysis, remaining teeth has shown a noteworthy link, possibility of the stated analysis confounded by age and/or years of education is to be considered. Further longitudinal investigations, including comprehensive clinical dental and brain-imaging information, are needed to shed further light on probable causal relationship(s) between dental status and cognition in adulthood and aging.

## Figures and Tables

**Table 1:** The table shows the relation of age, years of education, occupation, medical history, stress, and remaining teeth to cognitive impairment.

Variables	Age P value 0.000 (25.65)		Year of education P value 0.000 (131.28)				Occupation P value 0.000 (24.96)			Stress P value 0.139			Medical history P value 0.004 (32.87)							Remaining teeth P value 0.000 (54.63)		
Class interval	40-55	56-70	0-5	6-10	11-15	16-20	Nil	Teacher	Doctor	Mild	Moderate	High	Nil	HTN	DM	CVS	Thyroid	Arthritis	Asthma	0-16	17-28	>28

Mild	92.2%	7.8%	8.8%	22.1%	44.6%	24.5%	74%	23%	2.9%	47.5%	37.7%	14.7%	83.3%	6.4%	5.4%	1%	2.5%	1%	0.5%	2%	71.1%	27%

Moderate	69.3%	30.7%	60.2%	30.7%	6.8%	2.3%	97.7%	2.3%	0%	56.8%	33%	10.2%	58%	12.5%	19.3%	1.1%	1.1%	4.5%	3.4%	13.6%	81.8%	4.5%

Severe	87.5%	12.5%	100%	0%	0%	0%	10%	0%	0%	75%	0%	25%	75%	25%	0%	0%	0%	0%	0%	50%	50%	0%

**Table 2:** The table shows relation of cognitive variables with age.

Cognitive function	40-55 years	56-70 years	P value
Less Attention	45 (65.2%)	24 (34.8%)	*0.000*
More Attention	211 (91.3%)	20 (8.7%)	

Poor Recall	72 (75%)	24 (25%)	*0.000*
Good Recall	184 (90.2%)	20 (9.8%)	

Less Orientation	1 (50%)	1 (50%)	0.15
High Orientation	255 (85.6%)	43 (14.4)	

Low Registration	6 (100%)	0 %	0.305
High Registration	250 (85%)	44 (15%)	

Poor Language	3 (75%)	1 (25%)	0.556
Good Language	253 (85.5%)	43 (14.5%)	

**Table 3:** The table shows relation of cognitive variables with year of education.

Cognitive function	*0 - 5 Yrs.*	*6 – 10 Yrs.*	*11 -15 Yrs.*	*16 -20 Yrs.*	P value
	*(N =79*	*(N=72)*	*(N=97)*	*(N=52)*	
Less Attention	40 (58%)	22 (31.9%)	3 (4.3%)	4 (5.8%)	*0.000*
More Attention	39 (16.9%)	50 (21.6%)	94 (40.7%)	48 (20.8%)	

Poor Recall	48 (50%)	21 (21.9%)	15 (15.6%)	12 (12.5%)	*0.000*
Good Recall	31 (15.2%)	51 (25%)	82 (40.2%)	40 (19.6%)	

Poor Language	4 (100%)	0	0	0	*0.01*
Good Language	75 (25.3%)	72 (24.3%)	97 (32.8%)	52 (17.6%)	

Less Orientation	2 (100%)	0	0	0	0.131
High Orientation	77 (25.8%)	72 (24.2%)	97 (32.6%)	52 (17.4%)	

Low Registration	1 (16.7%)	1 (16.7%)	4 (66.7%)	0	0.302
High Registration	78 (26.5%)	71 (24.1%)	93 (31.65)	52 (17.7%)	

**Table 4:** The table shows relation of cognitive variables with occupation.

Cognitive function	*NIL*	*Teacher*	*Doctor*	P value
	*(N =245)*	*(N=49)*	*(N=6)*	
Less Attention	66 (95.7%)	3 (4.3%)	0	*0.003 *
More Attention	179 (77.5%)	46 (19.9%)	6 (2.6%)	

Poor Recall	89 (92.7%)	6 (6.3%)	1 (1%)	*0.003*
Good Recall	156 (90.2%)	43 (21.1%)	5 (2.5%)	

Poor Language	4 (100%)	0	0	0.63
Good Language	241 (81.4%)	49 (16.6%)	6 (2%)	

Low Registration	3 (50%)	3 (50%)	0	0.077
High Registration	242 (82.3%)	46 (15.6%)	6 (2%)	

Less Orientation	2 (100%)	0	0	0.798
High Orientation	243 (81.5%)	49 (16.4%)	6 (2%)	

**Table 5:** The table shows relation of cognitive variables with medical history.

Cognitive function	*Nil*	*HTN*	*DM*	*CVS*	*HYPO*	*RA*	*Ast.*	
	*(N =277)*	*(N=26)*	*(N=28)*	*(N=3)*	*(N=6)*	*(N=6)*	*(N=4)*	
Less Attention	37 (53.6%)	13 (18.8%)	17 (24.6%)	0	0	0	2 (2.9%)	*0.000*
More Attention	190 (82.3%)	13 (5.6%)	11 (4.8%)	3 (1.3%)	6 (2.6%)	6 (2.6%)	2 (0.9%)	

Poor Language	0 (65.2%)	2 (7.69%)	0	1 (25%)	1 (25%)	0	0	*0.000*
Good Language	227(76.7)	24 (92.3%)	28 (9.5%)	2 (0.7%)	5 (1.7%)	6 (2%)	4 (1.4%)	

Less Orientation	2 (100%)	0	0	0	0	0	0	0.996
High Orientation	225 (75.5%)	26 (8.7%)	28 (9.4%)	3 (1%)	6 (2%)	6 (2%)	4 (1.3%)	

Low Registration	6 (100%)	0	0	0	0	0	0	0.923
High Registration	221 (75.2%)	26 (8.8%)	28 (9.5%)	3 (1%)	6 (2%)	6 (2%)	4 (1.3%)	

Poor Recall	64 (66.7%)	8 (8.3%)	13 (13.5%)	2 (2.1%)	3 (3.1%)	4 (4.2%)	2 (2.1%)	0.099
Good Recall	163 (79.9%)	18 (8.8%)	15 (7.4%)	1 (0.5%)	3 (1.5)	2 (1%)	2 (1%)	

**Table 6:** The table shows relation of cognitive variables with stress level.

Cognitive function	*Mild*	*Moderate*	*Severe*	P value
	*(N =153)*	*(N=106)*	*(N=41)*	
Poor Language	1 (25%)	0	3 (75%)	*0.001*
Good Language	152(51.4%)	106 (35.8%)	38 (12.8%)	

Less Orientation	2 (100%)	0	0	0.380
High Orientation	151 (50.7%)	106 (35.6%)	41 (13.8%)	

Low Registration	1 (16.7%)	3 (50%)	2 (33.3%)	0.172
High Registration	152 (51.7%)	103 (35%)	39 (13.3%)	

Less Attention	38(55.1%)	25 (36.2%)	6 (8.7%)	0.381
More Attention	115 (49.8%)	81 (35.1%)	35 (15.2%)	

Poor Recall	52 (54.2%)	36 (37.5%)	8 (8.3%)	0.182
Good Recall	101 (49.5%)	70 (34.3%)	33 (16.2%)	

**Table 7:** The table shows relation of cognitive variables with remaining teeth.

Cognitive function	*≤ Half Dentation*	*17 -28 *	*> 28 (N=59)*	*P value *
	*(N=20)*	*(N=221)*		
Less Attention	17 (24.6%)	49 (71%)	3 (4.3%)	*0.000*
More Attention	3 (1.3%)	172 (74.5%)	56 (24.2%)	

Poor Recall	13 (13.5%)	75 (78.1%)	8 (8.3%)	*0.000*
Good Recall	7 (3.4%)	146 (71.6%)	51 (25%)	

Poor Language	2 (50%)	2 (50%)	0	*0.002*
Good Language	18 (6.1%)	219 (74%)	59 (19.9%)	

Less Orientation	0	2 (100%)	0	0.69
More orientation	20 (6.7%)	219 (73.5%)	59 (19.8%)	

High registration	1 (16.7%)	3 (50%)	2 (33.3%)	0.37
Low registration	19 (6.5%)	218 (74.1%)	57 (19.4%)	

**Table 8:** Frequency distribution table for variables related to medical & personal history, along with comparative evaluation of these variables with the MMSE score.

S. No.	Variable		Descriptive Statistics	Inferential Statistics
			N (%)	P value (chi-square Variate)
1.	Medical History	Nil	227 (75.7)	0.701 (3.818)
		Hypertension	26 (8.7)	
		Diabetes Mellitus	28 (9.3)	
		Cardiovascular	3 (1)	
		Hypothyroidism	6 (2)	
		Hyperthyroidism	0	
		Rheumatoid Arthritis	6 (2)	
		Asthma	4 (3)	

2	Stress	Mild (0–3)	153 (51)	0.10 (4.605)
		Moderate (4-7)	106 (35.3)	
		Severe (8–10)	41 (13.7)	

3	Remaining Teeth	≤ Half Dentation (0-16)	20 (6.7)	*0.000 (25.392)*
		17-28	221 (73.7)	
		> 28	59 (19.7)	

4	Age	40-55 Yrs.	256 (85.3)	0.861(0.031)
		56–70 Yrs.	44 (14.7)	

5	Year of Education	0–5 Yrs.	79 (26.3)	*0.000 (22.993)*
		6–10 Yrs.	72 (24)	
		11–15 Yrs.	97 (32.3)	
		16–20 Yrs.	52 (17.3)	

6	Occupation	Nil	245 (81.7)	0.397 (1.845)
		Teacher	49 (16.3)	
		Doctor	6 (2)	

**Table 9:** Binominal logistic regression analysis.

Predictor Variable	Odd Ratio	p value	95% Confidence Interval (CI) for EXP(B)	
			Lower limit	Upper limit
Age	1.046	0.381	0.9454	1.158
Stress	0.000	0.976	0.000	-
Years of Education	0.999	0.991	.792	1.259
Remaining Teeth	0.952	0.399	.850	1.067

## Data Availability

The data used to support the findings of this study are available from the corresponding author upon request.
